# Hypodermic Use of Morphia

**Published:** 1879-11-20

**Authors:** J. A. Houtz


### *HYPODERMIC USE OF MORPHIA.*

The request of Dr. Kane, of New York, to answer certain questions in regard to hypodermic injection of morphia, prompts me to give some of my experience in that direction in the past eight years. I do so the more readily because there seems to be a strong prejudice in the minds of some of the older country practitioners against the hyyodermic use of morphia. I was in consultation not long since with a very intelligent physician of great standing, and an army surgeon of the late war, who said he had never used the hypodermic syringe, and did not intend doing so, as he considered it dangerous and uncalled for. I am acquainted with a number of other regular physicians, who, either from want of appreciation of its great value, or thorough prejudice against it, do not own hypodermic syringes, and consequently never make use of it.

I was called upon a few years ago to take charge of a case of very acute uterine pains, in connection with great irritability of the stomach. I relieved her with two fourth-grain injections of sulph. morphia.

Now, it must be admitted that the hypodermic use of morphia is a matter not to be trifled with by any one, or tampered with by persons unacquainted with its proper use. But neither ought poisonous medicine, like arsenic, strychnia, etc., be carelessly or ignorantly used; and yet no one would think of discardtng these valuable remedies because they require care in their administration. That is one of our most potent means for the relief of human suffering, no one doubts who has had any experience in its use. In acute pains, of a spasmodic nature, is where its great value is most apparent; and, in fact, in many cases its use is almost indispensable.

In illustration of its great utility and its superiority over giving by mouth in particular cases, I will cite the history of a case of severe cramp of the stomach in a young lady I was called upon to treat about six months ago. There was nothing peculiar about the case, excepting the spasmodic closure of the cardiac orifice of the stomach, preventing the entrance of any substance whatever into the organ. I received the call on my way to another patient, and unfortunately did not have with me my hypodermic syringe; I gave her at once, by mouth, sulph. morphia, half a grain, to be repeated every hour until pain is relieved; I also ordered a large mustard plaster over the gastric region, to be followed by hot stupes; four hours after my first visit I was sent for again; I found the pain no better, though she had already taken two grains of morphia; I now injected, within three-fourths of an hour of each other, two fourth-grain doses of morphia, which relieved her entirely in twenty minutes after the last dose. I felt some anxiety after she was relieved as to the effects of the morphia taken by mouth when the stomach would become relaxed, but she recovered without much trouble.

Take as another instance, illustrative of its indispensable value, a severe case of cholera morbus, with excessive vomiting, in which nothing will be retained in the stomach sufficiently long to produce relief; a hypodermic injection of a third or a fourth of a grain of morphia will almost invariably relieve your patient in less time than it takes to send a prescription to the nearest apothecary. There are many other instances in which if the hypodermic syringe is not used the attendant does not do his full duty, up to the latest advances in medical scienee. I have frequently entirely relieved the urgent distress and suffering of cases of cholera morbus and colic in from ten to fifteen minutes, a result that cannot be accomplished in any other way. Of all the means at our disposal for the combatting of disease and suffering, I know of none in which I have such implicit confidence, as in the hypodermic syringe. When called to a case of acute pain, I feel certain I can relieve it, no matter whether my patient can retain anything in his stomach or not; and besides that I always know just how much morphia is in my patient's system, which is not always the case in giving by mouth. The evil results sometimes accompanying and following the hypodermic use of morphia can generally be avoided by care in performing the operation. The solution should be thorough and clear of extraneous matter, and I always heat it to about the temperature of the body before injecting; the greatest danger is in injecting into a vein sufficiently large to carry the whole dose at once into the circulation; that can be avoided by selecting a place where the large veins are least numerous, and by injecting, say a third of the dose and then waiting eight or ten seconds, when, if in a vein, the symptoms will show themselves. The first symptoms are a feeling of great fullness of the head and intense flushing of the face, coming on within a few seconds after the operation. Such, at least, was the case in a patient of mine.

Care should also be taken to introduce the point of the needle well into the subcutaneous cellular tissue, and not inject into the substance of the skin, otherwise the skin at once swells, as if stung by a bee, and abscesses are liable to follow. To do this properly the skin should be raised by pinching a fold of it between the thumb and finger and entering the needle perpendicularly to its surface, and not, as I have seen it done, at but a slight angle to the surface, passing through the skin for half an inch or more before entering the cellular tissue, causing considerable pain. I saw several abscesses follow its use, but in every instance I am satisfied the point of the needle had not been clearly in the subcutaneous cellular tissue. My usual dose is a fourth of a grain; less if the pain is slight, and more if extraordinarily severe; I have already given as much as three grains in a case of severe cramps in a person addicted to the excessive habitual use of opium.

I usually give morphia sulph. alone, excepting in neuralgic affections, when I combine a fourth grain morphia to a sixteenth of a grain of sulph. atropia. I do not submit this to your readers as embodying anything new or original, but merely as a gentle reminder to the inexperienced in this matter, of some interesting facts connected with its practical use.—*Dr. J. A. Houtz in Medical and Surgical Rep.*

